# The Association between Age-Related Changes in Oral Neuromechanics and Alzheimer’s Disease

**DOI:** 10.20900/agmr20210011

**Published:** 2021-04-27

**Authors:** Fritzie I. Arce-McShane

**Affiliations:** Department of Organismal Biology and Anatomy, University of Chicago, 1027 East 57th Street (Anatomy Suite 303),Chicago, IL 60637, USA

**Keywords:** orofacial sensorimotor cortex, motor control, somatosensation, neural network, chewing, mastication, swallowing, aging, Alzheimer’s Disease

## Abstract

The global population of 80 years and older is predicted to reach 437 million by 2050. As overall brain structure and function progressively degrades, older and younger adults show differences in sensorimotor performance and brain activity in the sensorimotor regions. Oral sensorimotor functions are an important area of focus in natural aging and Alzheimer’s Disease (AD) because oral health issues are commonly found in both elderly and AD populations. While human behavioral studies on changes in oral sensorimotor functions abound, very little is known about their neuronal correlates in normal and pathological aging.

## INTRODUCTION

Sensorimotor control of oral behaviors is complex and involves the integration of afferent information from and efferent commands to the tongue and jaw to effect functionally critical and highly coordinated movements of breathing, feeding and speech. Many age-related oral health problems, such as masticatory dysfunction, dysphagia, and tooth loss have been associated with Alzheimer’s Disease (AD) [1-12]. Diminished sensation, weakness of orofacial muscles, and impaired coordination, which accompany healthy aging, can cause difficulties in mastication and swallowing. With diminished sensation, the brain cannot sense the shape and position of the tongue relative to the teeth, information vital for detecting food properties when chewing and knowing when to swallow safely. How cortical and biomechanical (*“neuromechanical”*) changes in oromotor behavior contribute to the onset and progression of AD and age-related dementias (ARD) are widely unknown. This is largely because of a fundamental gap in understanding the neuromechanical processes at the level of large-scale activity of single neurons and neuronal networks that underlie *healthy aging*. This represents an important problem because until they are understood the cortical mechanisms underlying *pathological aging* in AD will remain largely incomprehensible.

## UNDERSTANDING THE BIOMECHANICS AND CORTICAL CONTROL OF OROFACIAL FUNCTIONS

Neurons in the primary motor (MIo) and somatosensory (SIo) areas of the orofacial cortex (a) modulate their activity during performance of orofacial tasks such as generating tongue protrusive force and bite force, (b) encode the direction and magnitude of tongue protrusive force, and (c) form coherent networks within and across these areas in a reciprocal manner, and (d) undergo learning-induced plasticity [13-16]. Currently, we are investigating the neural bases of oral somatosensation in the orofacial sensorimotor cortex in young non-human primates (NHPs) to dissociate the cortical representations of touch and proprioception during natural feeding behavior by using an innovative sequence of nerve blocks to the sensory branches of the trigeminal nerve, together with multi-electrode array recordings and 3D tracking of tongue and jaw movements. During feeding, a rich array of oral sensations is used to monitor bite forces, teeth contact, and the tongue moving and touching other oral structures (e.g., palate, teeth, gingiva). Because the location of the tongue inside the oral cavity makes it difficult to measure tongue movements, so are the sensations naturally occurring with these movements. Consequently, very little is known about cortical representations of these stimuli in the context of natural oromotor behavior. Tactile and proprioceptive signals to the tongue provide information about food properties, such as texture (soft, sticky, hard or grainy) or bolus size to regulate bite force, to predict when to swallow safely, and to perceive where the tongue is relative to other oral structures. Unlike the rest of the body, proprioceptive and tactile inputs to the tongue are anatomically distinct, with the former served by the hypoglossal nerve and the latter by the lingual nerve. I leveraged this unique anatomy to cleanly dissociate their cortical representations; by using an innovative sequence of local anesthetic blocks of trigeminal nerve sensory branches, tactile inputs are silenced while preserving proprioceptive inputs during feeding behavior. Moreover, there is a big challenge of high-resolution tracking of a wide array of tongue movements inside the oral cavity simultaneously with probing dynamic processes involving large populations of neurons across connected regions in behaving NHPs. Our laboratory has overcome these difficulties by using high resolution (>200 Hz) biplanar videoradiography and the X-ray Reconstruction of Moving Morphology (XROMM) (https://www.xromm.org) for precise tracking of tongue and jaw kinematics in 3D [17,18] while recording from large populations of neurons from multiple cortical regions (areas 3a, 3b, 1, 2, rostral and caudal MIo) [19,20]. These newly developed methods will help us understand the effects of aging on the critical functions served by the orofacial system that are vulnerable to sensorimotor decline.

**Figure 1.**
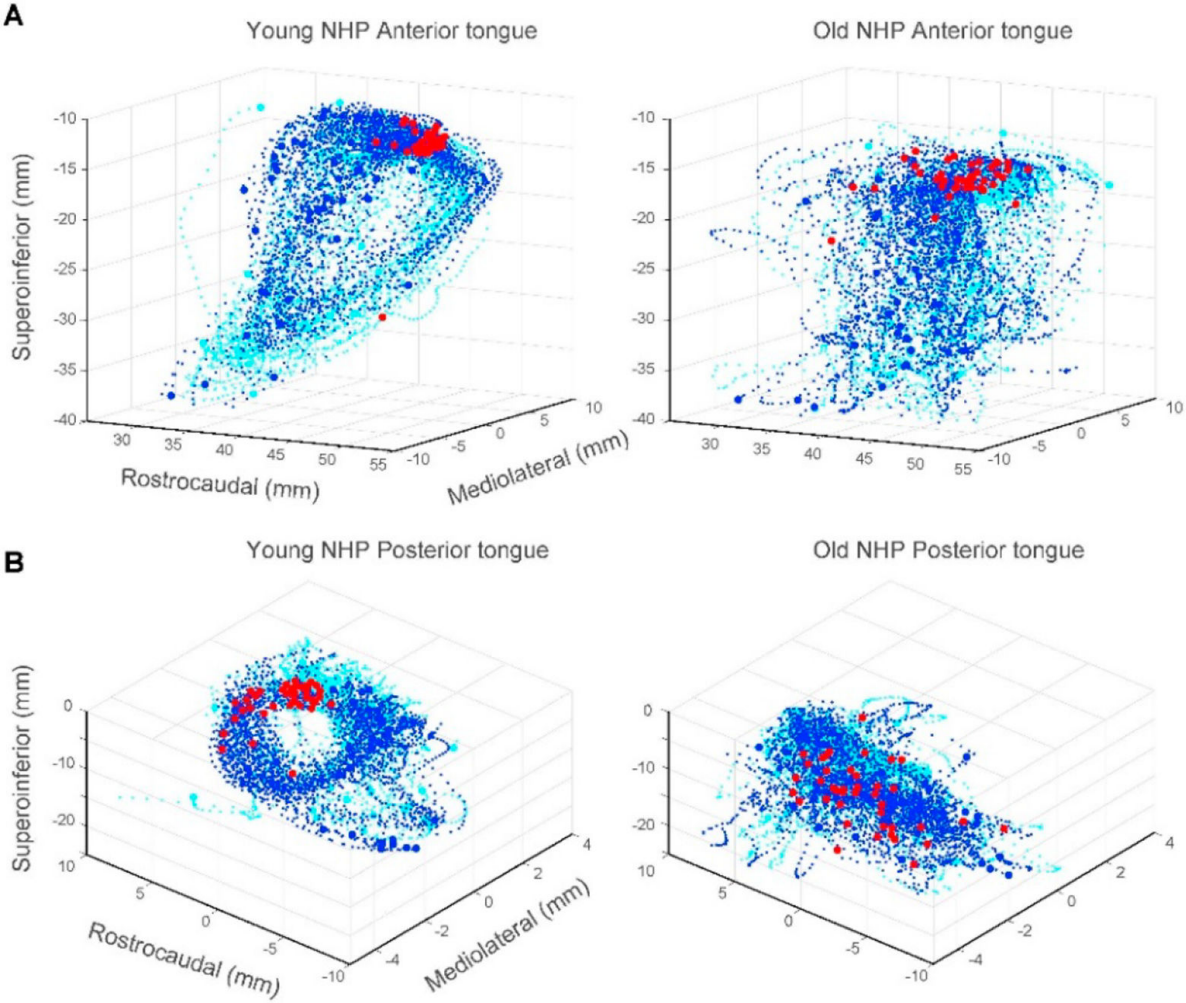
Age-related changes in tongue kinematics during swallows. (**A**) Anterior tongue trajectories ±0.5 s around swallows were stereotypical and cyclic in the young NHP (left) but not in the old NHP (right). Swallows occurring around minimum gape (red circles) were more tightly clustered in the young NHP. Blue and cyan circles denote start and end of tongue trajectories. Blue and cyan dots denote tongue trajectories 0.5 s before and after swallows, respectively. (**B**) As in (A), shown for trajectories of the posterior region of the tongue in the young vs old NHP.

The sensorimotor changes found in healthy elderly population include difficulties in mastication and swallowing, diminished sensation, weakness of orofacial muscles, slowness of movement, and impaired coordination [21-28]. Neuroimaging studies found effects of aging on brain activation and functional connectivity in sensorimotor regions at resting state [29] and related to chewing and swallowing [30-33]. With diminished sensation, the brain cannot sense the position of the tongue relative to the jaw or teeth nor the force applied to the teeth when chewing. Similarly, the encoding of the amount and direction of bite force is impaired following tooth loss owing to the removal of mechanoreceptors in the periodontal ligament [34,35]. Indeed, edentulous older patients with dentures show limited activation of brain regions typically associated with teeth clenching [28]. Preliminary findings from our laboratory are consistent with dysfunctions in mastication and swallowing found in the elderly (Figure 1); we found marked differences in tongue and jaw kinematics around chews and swallows in young vs. old NHPs during feeding [36]. The complex control of chewing and swallowing involves multiple cortical and subcortical regions, including limbic and prefrontal regions of the cortex [30-33]. Cortical regions that may exert cognitive-affective influences on oral sensorimotor functions may underlie the potential association with oral dysfunctions found in AD/ARD.

How do these neuromechanical changes found in healthy aging differ from those found in pathological aging such as in AD? A better understanding of cortical processes underlying sensorimotor decline in healthy and pathological aging will require investigating the dynamic processes involving large populations of neurons across connected regions (e.g., prefrontal, parietal, and sensorimotor) in behaving animal models. Specifically, by contrasting neuromechanical changes related to healthy-aging with those found in individuals with AD, we may be able to identify individuals at risk for developing AD or those who may be in the prodromal stage of the disease. Understanding how these biomarkers may serve as early signatures of AD could be helpful in providing early diagnosis and intervention, thus delaying AD progression and reducing the severity of debilitating oromotor dysfunctions prevalent in AD/ARDs. Indeed, the early intervention MEND™ protocol demonstrated reversal of memory loss in prodromal AD patients by avoiding risk factors [37]. In addition, a diagnostic tool similar to the one used for motoric cognitive risk syndrome [38] could include oromotor deficits to identify individuals at higher risk of dementia.

## THE LINK BETWEEN AGE-RELATED OROMOTOR DYSFUNCTION AND AD

While the pathophysiological link between oromotor dysfunction and AD is still unknown, there are strong indications from the literature that the two may be related, but do not suggest a causal relationship: (1) *Oral health and memory may influence each other* (see reviews by [6,9,39,40]). Decrease in masticatory activity, due to a soft diet or loss of teeth, causes memory loss and neuronal degeneration in mice [41,42]. Mastication improves cerebral blood flow, which in turn improves memory functions in humans [30,43]. In elderly people with full dentures, but not in those with full natural teeth, 22% of executive functions were predicted by complaints of the masticatory system and 19% of episodic memory was predicted by masticatory performance [44]. It has been suggested that the relationship between mastication and memory becomes more prominent when mastication is reduced due to tooth loss or oral pain. On the other hand, one should also consider the person’s ability to adapt mastication to changes in dental status [45] and whether this is impaired in AD/ARD. Currently, evidence supporting the association between tooth loss, masticatory performance, and dementia is still lacking [46,47]. (2) *AD and oromotor dysfunctions share common risk factors*: aging, diet/nutrition, and socio-economic status [7]. Several longitudinal studies showed the prevalence of oromotor dysfunction, especially dysphagia, in patients with AD/ARD [4,9,11,40]. Early-stage AD patients without overt signs of dysphagia already showed lower BOLD response in the swallowing cortical network [48]. More importantly, participants with the fewest teeth had the highest risk of prevalence and incidence of dementia [10]. (3) *Porphyromonas gingivalis* and *gingipains* in chronic periodontitis (gum disease) were identified in the brain of AD patients, and levels of gingipains were correlated with tau and ubiquitin pathology [2]. In severe periodontal disease, periodontal tissues, including alveolar bone and periodontal ligament, are destroyed and lead to the loosening and loss of teeth, which in turn may cause masticatory dysfunctions. Periodontal disease has been found to be associated with poor cognitive performance [49-51].

## CONCLUSIONS

In the advanced stage, AD’s devastating effects on the quality of patient’s life and the burden on the caregiver are further heightened. It is therefore expedient to identify potential contributing factors to the onset and progression of AD/ARD. Determining whether oromotor dysfunction could be identified as a *risk factor* to the development of the sporadic form of AD and/or serve as an *early diagnostic tool*. Thus, the early identification of individuals with chronic oral health issues at risk for developing AD and the development of effective interventions to enhance oral health outcomes in this group may aid in preventing the onset or allay the progression of AD/ARD in these populations.
